# Risk Factors of Vitamin D Deficiency in Chinese Ischemic Stroke Patients: A Cross-Sectional Study

**DOI:** 10.3389/fnagi.2020.613498

**Published:** 2021-01-18

**Authors:** Hanpei Miao, Hanyu Zhu, Xiaoqian Luan, Guiqian Huang, Meixia Chen, Zhengzhong Yuan, Zhen Wang

**Affiliations:** ^1^Department of Ophthalmology, Wenzhou Medical University, Wenzhou, China; ^2^Department of Neurology, The First Affiliated Hospital of Wenzhou Medical University, Wenzhou, China; ^3^Department of Neurology, The Yuhuan People’s Hospital, Taizhou, China; ^4^Department of Traditional Chinese Medicine, The First Affiliated Hospital of Wenzhou Medical University, Wenzhou, China

**Keywords:** ischemic stroke, vitamin D, fibrinogen, National Institute of Health Stroke Scale score, Chinese

## Abstract

**Purpose:**

Lower serum vitamin D has been reported to be associated with stroke. This study aimed to analyze the risk factors of vitamin deficiency in Chinese stroke patients, and further analyze its impact in different gender and their clinical variables.

**Methods:**

982 stroke patients were enrolled. Laboratory parameters such as serum vitamin D, apolipoprotein A-I (ApoA-I), apolipoprotein B (ApoB), ApoA-I/ApoB, cholesterol (CH), fibrinogen (FIB), blood glucose (Glu), high-density lipoprotein (HDL), low-density lipoprotein cholesterol (LDL-C), and triglyceride (TG) were collected and recorded. The severity of stroke was assessed by National Institute of Health Stroke Scale (NIHSS) score. Based on their serum vitamin D level, patients were divided into three groups: Vitamin D deficiency (<50 nmol/L), vitamin D insufficiency (≥50–75 nmol/L) and vitamin D sufficiency (≥75 nmol/L) and differences were compared among the three groups. Statistical analyses were done to assess the risk factors for serum vitamin D deficiency in our ischemic stroke patients.

**Results:**

Gender, NIHSS, and FIB showed significant differences among the vitamin D groups (*P* < 0.001 ∼ *P* = 0.002). The female gender (OR = 2.422, *P* < 0.001), severity of stroke using NIHSS (OR = 1.055, *P* = 0.008) and FIB (OR = 1.256, *P* = 0.005) were risk factors of vitamin D deficiency in ischemic stroke patients. In subgroup analysis, NIHSS was significantly associated with vitamin D deficiency in the male group (OR = 1.087, *P* = 0.002) and higher FIB group (OR = 1.078, *P* = 0.001).

**Conclusions:**

The female gender, severity of stroke using NIHSS and FIB were risk factors for vitamin D deficiency in our incident stroke patients. NIHSS was more sensitive to vitamin D deficiency in male ischemic stroke patients. Besides, under higher FIB circumstance, the increasing NIHSS score was more related to the vitamin D deficiency. Levels of vitamin D in patients with ischemic stroke should be well monitored during the disease cascade.

## Introduction

Recent reports have shown a major rise in the aging population worldwide especially in China, the country with the largest population. Stroke has been reported to be one of the common cause of death worldwide but has been shown to be the leading cause of death in China (Zhou et al., 2019). Zhou et al. (2019) showed that more 80% of death cases in China were due to stroke. Nonetheless, reports of stroke in the Chinese population were neither population based or of reduced range and diagnostic precision; additionally most of these reports were done over two decades ago. Moreover, China has experienced rapid changes in their sociodemographic and health transitions have shown a significant impact on the prevalence of risk factors associated with stroke (Yang et al., 2013). Statistics have shown that there’s been a significant rise in the occurrence of smoking, hypertension and diabetes, all of which have been reported to be risk factors for stroke (Feigin et al., 2016). With these rapid changes in China, it would be helpful to obtain an up to date and accurate risk factor(s) to help in the recently implemented therapies and prevention measures.

Vitamin D, also known as 25-hydroxyvitamin D [25(OH) D] is known for its effects on the metabolism on bones and homeostasis of calcium (Berghout et al., 2019). A meta-analysis suggested that vitamin D helps reduces hazard of cardiovascular diseases and cognitive disorders, shedding light on its non-skeletal effects on the body (Zhou R. et al., 2018). A previous report showed the neuro-protective effects of vitamin D and its effect on the integrity of the blood brain barrier (Theodoratou et al., 2014). Since deficiency in vitamin D can be treated, vitamin D may have an important public health inference in the prevention of age-related neurodegenerative diseases such as stroke, especially in this part of the world, China.

Recent reports on the association between vitamin D and its risk on stroke have been inconclusive. A report showed that insufficient levels of vitamin D increases the risk of stroke (Zhou R. et al., 2018) while some reports showed no effect of low levels vitamin D as a risk factor of stroke (Perna et al., 2013; Skaaby et al., 2014). Nonetheless, it has been reported that 78% of patients with ischemic stroke have vitamin D deficiency in China (Si et al., 2019). Due to the inconsistent reports, we aimed to assess the association of vitamin D deficiency with ischemic stroke in a Chinese population (Han race). We also aimed to analyze the risk factors of vitamin D deficiency in stroke patients using different models.

## Materials and Methods

### Demographics

This study was conducted at The First Affiliated Hospital of Wenzhou Medical University. Patients were enrolled from a retrospective database which included sequential patients who were admitted and diagnosed at the Neurology Department. Patients were enrolled within 24 h after ischemic stroke between 2017 and 2020. The ethics of committee of The First Affiliated Hospital of Wenzhou Medical University approved of the study and it followed the declaration of Helsinki. All participants provided informed written consent.

All patients underwent cerebral computed tomography (CT) or magnetic resonance imaging (MRI) to confirm the diagnosis ischemic stroke within 24 h of admission. The exclusion criteria were the following: 1. Confirmed diagnosis of transient ischemic attack (TIA); 2. Had preventive antibacterial treatment; 3. Had an infection 2 – 3 weeks before admission; 4. History of neurodegenerative disease such as Alzheimer’s disease, Parkinson’s disease, cerebral hemorrhage, and brain trauma; 5. Incomplete medical information; 6. Severe hepatic disease; 7. Severe renal disease; and 8. Patient was on vitamin D supplement before occurrence of ischemic stroke or has osteoporosis.

### Assessing the Concentration of Vitamin D

Concentration levels of vitamin D was done using an competitive electrochemiluminescence protein binding assay (COBAS Roche Diagnostic GmbH, Germany) as previously reported (Huang et al., 2019). Blood samples were taken within 24 h of admission. Patients were vitamin D levels less than 50 nmol/L were described as being vitamin D deficient (Park et al., 2015; Zainel et al., 2019).

### Other Clinical Characteristics and Laboratory Parameters

All patients’ demographics and clinical information were collected and recorded. Clinical information such as history of hypertension, diabetes, systolic (SBP) and diastolic (DBP) blood pressures were collected; classification of stroke was done according to TOAST criteria (Arsava et al., 2017). Other clinical parameters included blood routine examination to assess apolipoprotein A-I (ApoA-I), apolipoprotein B (ApoB), ApoA-I/ApoB, cholesterol (CH), fibrinogen (FIB), fasting blood glucose (Glu), high-density lipoprotein (HDL), low-density lipoprotein cholesterol (LDL-C), and triglyceride (TG). Severity of stroke was assessed using National Institute of Health Stroke Scale (NIHSS) score (Kwah and Diong, 2014) within 24 h of admission.

### Statistical Analyses

All statistical analyses were performed using SPSS software version 22, SPSS Inc., Chicago, IL, United States. Descriptive analyses were used to compare participant characteristics across the categories of vitamin D. Vitamin D categories were classified using the following as previously reported: sufficient was defined as vitamin D greater than or equal to 75 nmol/L; insufficiency was defined as vitamin D greater or equal to 50 and less than 75 nmol/L; deficiency was defined as vitamin D less than 50 nmol/L. Continuous variables were described by mean (standard deviations), and frequencies and percentages were used to describe categorical variables. Statistical analyses among the three groups were done with one-way analysis of variance (ANOVA) or Kruskal–Wallis in continuous variables. Pearson’s chi-square or Fisher’s exact tests were used where appropriate. The clinical characteristics and laboratory parameters associated to the NIHSS was confirmed by univariate and multivariate linear regression analysis (the factors which showed *P* < 0.05 were used to create a multivariate model) and univariate logistic regression was used to predict risk factors for vitamin D deficiency in stroke patients. The significant factors for the univariate analysis were further selected for further multiple logistic regression models to predict the occurrence of vitamin D deficiency. Furthermore, all patients were divided to two group depending on gender and FIB level (Low FIB < 3.18, high FIB ≥ 3.18) (Zhou X. et al., 2018). Univariate analysis was used to analyze the association between the significant risk factors and vitamin D deficiency, and the multiple logistic regression was further used to adjust for potential confounding or risk factors. *P*-values less than 0.05 were considered statistically significant.

## Results

### Baseline Characteristics of the Patients Base on the Vitamin D Level

In this study, we enrolled a total of 982 ischemic stroke patients from the Neurology Department of The First Affiliated Hospital of Wenzhou Medical University between 2017 and 2020 [629 males (63.3%), 357 female (36.0%); mean age was 66.2 ± 11.7 years; Table 1]. Based on the vitamin D level, 295 patients were grouped as vitamin D deficiency (vitamin D level = 37.38 ± 9.12 nmol/L; Table 1), 422 patients were vitamin D insufficiency (vitamin D level = 61.64 ± 6.71 nmol/L; Table 1) and 265 patients were vitamin D sufficiency (vitamin D level = 91.83 ± 14.86 nmol/L; Table 1). The percentage of patients with vitamin D deficiency was 295 (30.0%); 155 (15.8%) males, and 140 (14.3%) females were vitamin D deficient. The clinical information and differences among the three groups using the vitamin D levels are shown in Table 1.

**TABLE 1:** Clinical data of enrolled patients.

**Variable**	**Total**	**Vitamin D deficiency (<50 nmol/L) *N* = 295**	**Vitamin D insufficiency (≥50–75 nmol/L) *N* = 422**	**Vitamin D sufficiency (≥75 nmol/L) *N* = 265**	***P*-value**
Gender, Female, *n* (%)	357 (36.0)	140 (47.5)	165 (39.1)	52 (19.6)	**<0.001**
Age, years	66.2 (11.74)	66.09 (13.21)	65.41 (11.17)	67.86 (10.59)	**0.027**
SBP, mm Hg	152.87 (24.37)	154.36 (24.16)	152.02 (23.88)	152.60 (25.37)	0.439
DBP, mm Hg	83.31 (13.80)	83.54 (13.81)	83.28 (13.81)	82.98 (14.02)	0.891
Vit D (nmol/L)	62.50 (22.94)	37.38 (9.12)	61.64 (6.71)	91.83 (14.86)	**<0.001**
HCY (μmol/L)	13.71 (8.34)	13.70 (5.82)	13.41 (8.53)	14.26 (10.26)	0.442
NIHSS score	4.14 (4.18)	4.97 (4.52)	3.71 (3.77)	3.91 (4.28)	**0.002**
Diabetes mellitus, n (%)	422 (42.7)	150 (35.6)	181 (43.0)	90 (21.4)	**0.001**
Hypertension, n (%)	725 (73.4)	222 (30.7)	307 (42.5	194 (26.8)	0.743
ApoA-I (mmol/L)	1.21 (0.25)	1.17 (0.24)	1.22 (0.24)	1.25 (0.26)	**<0.001**
ApoB (mmol/L)	0.85 (0.25)	0.87 (0.25)	0.83 (0.25)	0.84 (0.25)	0.218
ApoA-I/ApoB	1.55 (0.55)	1.45 (0.48)	1.59 (0.57)	1.62 (0.57)	**<0.001**
CH (mmol/L)	4.72 (1.25)	4.70 (1.23)	4.70 (1.27)	4.76 (1.17)	0.803
FIB (g/L)	3.64 (1.15)	3.91 (1.28)	3.49 (1.10)	3.56 (1.03)	**<0.001**
GLU (mmol/L)	6.46 (2.79)	6.66 (2.97)	6.57 (2.87)	6.05 (2.36)	**0.020**
HDL (mmol/L)	1.02 (0.26)	0.98 (0.25)	1.02 (0.25)	1.08 (0.27)	**<0.001**
LDL-C (mmol/L)	2.70 (0.93)	2.73 (0.90)	2.67 (0.94)	2.70 (0.91)	0.623
TG (mmol/L)	1.75 (1.06)	1.78 (1.11)	1.82 (1.15)	1.60 (0.80)	**0.023**
Stroke etiology, n (%)					0.835
Atherosclerosis	772 (78.1)	226 (29.4)	332 (43.2)	211 (27.4)	
Cardioembolism	127 (12.9)	42 (33.2)	52 (40.9)	33 (26.0)	
Small vessel occlusion	63 (6.4)	17 (27.4)	29 (46.8)	16 (25.8)	
Other causes	19 (1.9)	8 (42.1)	6 (31.6)	5 (26.3)	

SBP, systolic blood pressure; DBP, diastolic blood pressure; ApoA-I, apolipoprotein A-I; ApoB, apolipoprotein B; CH, cholesterol; FIB, fibrinogen; Glu, fasting blood glucose; HDL, high-density lipoprotein; LDL-C, low-density lipoprotein cholesterol; TG, triglyceride; NIHSS, Severity of stroke was assessed using National Institute of Health Stroke Scale score. * Continuous variables were described by mean (standard deviations), and frequencies (percentages) were used to describe categorical variables. Continuous variables were compared between the groups by the one-way analysis of variance (ANOVA) or Kruskal–Wallis test. Pearson’s chi-square test or Fisher’s exact tests was used for categorical variables.

### The Risk Factors Associated With NIHSS in All Stroke Patients

In a univariate linear regression analysis, age, SBP, DBP, vitamin D, ApoB, ApoA-I/ApoB, FIB LDL-C, and TG were significantly associated to NIHSS (*P* < 0.001 ∼ 0.049; Table 2), respectively. Parameters with significant associations were then put into a multivariate linear regression model; age, DBP, vitamin D, FIB, and TG showed significant association with NIHSS (*P* < 0.001 ∼ 0.009; Table 2), respectively.

**TABLE 2:** Factors associated with NIHSS in all stroke patents.

**Variable**	**Univariate Analysis**		**Multivariate Analysis**	
	**β (95%CI)**	***P***	**β (95%CI)**	***P***
Gender	−0.035(−0.919*to*0.310)	0.331		
Age, y	0.162(0.032*to*0.082)	**<0.001**	0.162(0.031*to*0.085)	**<0.001**
SBP	0.083(0.002*to*0.026)	**0.021**	−0.005(−0.015*to*0.013)	0.910
DBP	0.071⁢(-7.400×10⁢to-4⁢0.044)	**0.049**	0.109(0.009*to*0.060)	**0.009**
Vit D	−0.092(−0.029*to*−0.004)	**0.011**	−0.094(−0.030*to*−0.004)	**0.008**
HCY	0.058(−0.007*to*0.065)	0.114		
Diabetes mellitus	−0.563(−1.260*to*0.134)	0.113		
ApoA-I	−0.020(−1.538*to*0.876)	0.590		
ApoB	0.092(0.342*to*2.662)	**0.011**	0.087(−1.260*to*4.165)	0.293
ApoA-I/ApoB	−0.092(−1.210*to*−0.154)	**0.011**	−0.002(−0.811*to*0.786)	0.976
CH	0.061(−0.032*to*0.443)	0.089		
FIB	0.208(0.501*to*1.002)	**<0.001**	0.164(0.342*to*0.871)	**<0.001**
GLU	0.040(−0.047*to*0.166)	0.271		
HDL	0.037(−0.535*to*1.721)	0.302		
LDL-C	0.103(0.147*to*0.775)	**0.004**	0.022(−0.512*to*0.718)	0.743
TG	−0.138(−0.828*to*−0.270)	**<0.001**	−0.141(−0.869*to*−0.251)	**<0.001**

SBP, systolic blood pressure; DBP, diastolic blood pressure; ApoA-I, apolipoprotein A-I; ApoB, apolipoprotein B; CH, cholesterol; FIB, fibrinogen; Glu, fasting blood glucose; HDL, high-density lipoprotein; LDL-C, low-density lipoprotein cholesterol; TG, triglyceride; NIHSS, Severity of stroke was assessed using National Institute of Health Stroke Scale score. *Significant correlation (*P* < 0.05) between other parameters and NIHSS score using simple and multivariate linear regression analysis.

### The Risk Factors of Vitamin D Deficiency in All Patients

In a univariate binary logistic regression analysis, female, NIHSS, diabetes mellitus, and FIB were risk factors of vitamin D deficiency in all patients (OR = 1.066 ∼ 1.584, P ≤ 0.001; Table 3), while the ApoA-I, ApoA-I/ApoB and HDL were protective factors (OR = 0.331 ∼ 0.569, *P* < 0.001; Table 3). In a multivariate logistic regression model analysis, female (OR = 2.422, *P* < 0.001; Table 3), NIHSS (OR = 1.005, *P* = 0.008; Table 3) and FIB (OR = 1.256, *P* = 0.005; Table 3) showed greater significant risk of predicting vitamin D deficiency in stroke patients.

**TABLE 3:** Univariate and multivariate logistic regression analysis for vitamin D deficiency occurrence in all patients.

**Variable**	**Univariate Analysis**				**Multivariate Analysis**			
	**β Coefficient**	**OR**	**95%CI**	***P***	**β Coefficient**	**OR**	**95%CI**	***P***
Gender								
Male	Reference							
Female	0.671	1.956	1.479 to 2.587	**<0.001**	0.885	2.422	1.691 to 3.471	**<0.001**
Age, y	–0.002	0.998	0.986 to 1.010	0.742				
SBP	0.004	1.004	0.998 to 1.009	0.212				
DBP	0.002	1.002	0.992 to 1.012	0.694				
NIHSS	0.064	1.066	1.028 to 1.105	**0.001**	0.054	1.055	1.014 to 1.098	**0.008**
Diabetes mellitus	0.460	1.584	1.203 to 2.085	**0.001**	0.304	1.356	0.963 to 1.907	0.081
Hypertension	0.121	1.129	0.825 to 1.545	0.448				
HCY	4.970 × 10^–4^	1.000	0.983 to 1.016	0.954				
ApoA-I	–1.106	0.331	0.184 to 0.596	**<0.001**	–0.652	0.521	0.133 to 2.037	0.349
ApoB	0.476	1.609	0.934 to 2.774	0.087				
ApoA-I/ApoB	–0.565	0.569	0;0.430 to 0.752	**<0.001**	–0.217	0.805	0.555 to 1.167	0.253
CH	–0.017	0.984	0.880 to 1.099	0.771				
FIB	0.307	1.360	1.205 to 1.534	**<0.001**	0.228	1.256	1.072 to 1.472	**0.005**
GLU	0.035	1.036	0.988 to 1.086	0.142				
HDL	–1.000	0.368	0.210 to 0.646	**<0.001**	–0.922	0.398	0.114 to 1.390	0.149
LDL-C	0.064	1.066	0.920 to 1.236	0.394				
TG	0.044	1.045	0.920 to 1.185	0.499				

SBP, systolic blood pressure; DBP, diastolic blood pressure; ApoA-I, apolipoprotein A-I; ApoB, apolipoprotein B; CH, cholesterol; FIB, fibrinogen; Glu, fasting blood glucose; HDL, high-density lipoprotein; LDL-C, low-density lipoprotein cholesterol; TG, triglyceride; NIHSS, Severity of stroke was assessed using National Institute of Health Stroke Scale score. *Significant correlation (*P* < 0.05) based on univariate and multivariate logistic regression analysis for vitamin D deficiency.

### The Risk Factors of Vitamin D Deficiency in Female Group and Male Group

Table 4 shows the risk factors which were inclined to vitamin D deficiency in different gender groups. In a univariate logistic regression analysis, FIB significantly associated with vitamin D deficiency in both gender (female: OR = 1.476, *P* = 0.001; male: OR = 1.328, *P* < 0.001; Table 4). NIHSS was associated with vitamin D in male (OR = 1.087, *P* = 0.002; Table 4), and significant difference still remained after adjusting for risk factors.

**TABLE 4:** Univariate and multivariate logistic regression analysis for vitamin D deficiency occurrence in gender groups.

**Variable**	**Univariate analysis**				**Model 1**				**Model 2**			
	**β**	**OR**	**95%CI**	***P***	**β**	**OR**	**95%CI**	***P***	**β**	**OR**	**95%CI**	***P***
Female												
FIB	0.389	1.476	1.175 to 1.853	**0.001**	0.416	1.517	1.129 to 2.037	**0.006**				
NIHSS	0.051	1.052	0.995 to 1.113	0.074					0.027	1.027	0.965 to 1.093	0.402
Male												
FIB	0.284	1.328	1.148 to 1.537	**<0.001**	0.321	1.378	1.142 to 1.663	**0.001**				
NIHSS	0.071	1.073	1.023 to 1.126	**0.004**					0.083	1.087	1.030 to 1.147	**0.002**

FIB, fibrinogen; NIHSS, severity of stroke was assessed using National Institute of Health Stroke Scale score. *Significant correlation (*P* < 0.05) based on univariate and multivariate logistic regression analysis for vitamin D deficiency. Model 1 adjust age, diabetes mellitus, Hypertension, NIHSS; Model 2 adjust age, diabetes mellitus, Hypertension, FIB.

### The Risk Factors of Vitamin D Deficiency in Different FIB Level

In a univariate logistic regression, female showed significant risk implications in both FIB level (lower FIB: OR = 2.124, *P* = 0.003; higher FIB: OR = 1.722, *P* = 0.002; Table 5), while increasing NIHSS was a risk factor in higher FIB level (OR = 1.067, *P* = 0.003; Table 5). After adjusting for risk factors, Female (lower FIB: OR = 2.075, *P* = 0.015; higher FIB: OR = 1.807, *P* = 0.004; Table 5), NIHSS (higher FIB: OR = 1.078, *P* = 0.001; Table 5) showed the same results aforementioned in different FIB level.

**TABLE 5:** Univariate and multivariate logistic regression analysis for vitamin D deficiency occurrence in different FIB levels.

**Variable**	**Univariate analysis**				**Model 1**				**Model 2**			
	**β**	**OR**	**95%CI**	***P***	**β**	**OR**	**95%CI**	***P***	**β**	**OR**	**95%CI**	***P***
Low FIB												
Gender												
Male	**Reference**											
Female	0.753	2.124	1.289 to 3.499	**0.003**	0.730	2.075	1.152 to 3.738	**0.015**				
NIHSS	0.045	1.046	0.974 to 1.123	0.219					0.058	1.060	0.984 to 1.142	0.126
Higher FIB												
Gender												
Male	**reference**											
Female	0.543	1.722	1.224 to 2.422	**0.002**	0.591	1.807	1.205 to 2.708	**0.004**				
NIHSS	0.065	1.067	1.022 to 1.113	**0.003**					0.075	1.078	1.031 to 1.128	**0.001**

FIB, fibrinogen; NIHSS, severity of stroke was assessed using National Institute of Health Stroke Scale score. *Significant correlation (*P* < 0.05) based on univariate and multivariate logistic regression analysis for vitamin D deficiency. Model 1 adjust age, diabetes mellitus, Hypertension, NIHSS; Model 2 adjust age, gender, diabetes mellitus, Hypertension.

## Discussion

Our current study showed that deficiency of vitamin D is associated with severity of stroke using NHISS in our Chinese ischemic stroke patients. We also showed that the risk factors of vitamin D deficiency in stroke patients were the female gender, severity of stroke using NHISS and FIB.

Our results showed an association between vitamin D deficiency and severity of incident stroke suggesting that low levels of serum vitamin D may be a consequence of stroke. It has been reported that low vitamin D principally may be associated with both non-fatal and terminal stroke (Tu et al., 2014). Our current report is congruent with previous reports which showed that vitamin D deficiency is associated with the severity of ischemic stroke (Tu et al., 2014; Li et al., 2018). Reports on the association between vitamin D and stroke have been varying due to several explanations (Skaaby et al., 2014; Zhou R. et al., 2018). To start with, most studies used different cut-off values of serum 25(OH) D in their analyses and this may have swayed their findings. A study used below 45 nmol/L which did not yield a substantial relationship while another study used less than 35 nmol/L which led to significant results (Schneider et al., 2015). Our current study used less than 50 nmol/L as deficient vitamin D and found a significant association with the severity of incident stroke. Although the cutoff values for deficiency in vitamin D used in previous reports vary, our current report suggests less than 50 nmol/L used in the Chinese population may be of significance in the clinical field and a sufficient level of 75 nmol/L may be ideal for health benefits which is congruent with a previous report (Holick et al., 2011).

Vitamin D has been reported to stimulate dilation of vessels, reduce arterial pressure and recuperate blood flow post stroke to neurons by increasing the effect of nitric oxide synthase (NOS) (Yalbuzdag et al., 2015; Nie et al., 2017). Low and deficient levels of vitamin D has been reported to lead to vascular stiffness (Suthar et al., 2018). Moreover, deficient levels of vitamin D leads to narrower and stiffer vessels which increases the risk occlusion; besides, deficient levels of vitamin D in patients with ischemic stroke has been reported to be a risk factor for deep venous thrombosis (Wu and He, 2018). Furthermore, very low levels of vitamin D in ischemic stroke patients has been reported to be associated with severe white matter microstructural damage and cerebral small-vessel disease burden seen on MRI (Feng et al., 2019). As such, the association between deficiency of vitamin D and severity of stroke seen in our patients indicates that the lower the vitamin D levels the more severe the ischemic stroke patient is. Our report urges clinicians to pay close attention to the levels of vitamin D in patients with ischemic stroke to reduce worsen patient’s condition.

Several reports have shown on the gender association between vitamin D deficiency and stroke (Sun et al., 2012; Hu et al., 2019). A previous Swedish study showed that females with less than 50 nmol/L had a higher risk for stroke and morbidity (Leu Agelii et al., 2017). Another study also showed that females with less than 50 nmol/L of serum vitamin D had a moderate risk increase for stroke (Sun et al., 2012). On the other hand, a report found that deficiency in vitamin D increased the risk for stroke in males (Bajaj et al., 2014). A Japanese study also showed that males with vitamin D deficiency had a slight increase in the risk for stroke (Kojima et al., 2012, 2014). Our current study showed that vitamin D deficiency as risk factor in females was severe than that in males which is congruent with previous stroke reports done on middle-aged stroke patients (Kojima et al., 2012; Leu Agelii et al., 2017); however, the reason(s) behind this is still vague. A review reported that males are at a greater risk of stroke and vascular diseases when compared to females; the authors suggested that this may be due to the impact of androgens and estrogens (Abi-Ghanem et al., 2020) in the body. Another research showed that females had a lower risk of stroke incidence in 45–74 years old when compared to the male gender (Jiang et al., 2020). Our report suggests that the risk of vitamin D deficiency in ischemic stroke patients is more significant in the female gender. Further studies with longitudinal reports are needed to elucidate this.

It has been reported that ischemic stroke triggers an acute phase response in the vascular system resulting in an increase of circulating inflammatory markers (Aleva et al., 2020). The initiation of tissue injury in the cerebral vessels triggers inflammatory response which triggers upregulation of hepatic fibrinogen and recruits coagulation cascade. High concentrations of fibrinogen in acute stroke patients have been reported to be a response to brain damage and the underlying vessel wall disease (Rothwell et al., 2004; Di Napoli and Singh, 2009). Our current study showed that high serum fibrinogen in acute ischemic stroke patients is a risk factor for vitamin D deficiency. We also suggest that high concentration of fibrinogen (which translates as increase in the activation of platelets) may be a risk factor for low serum concentrations of vitamin D less than 50 nmol/L in ischemic stroke patients. To the best of our knowledge, this is the first study to report on high concentration of fibrinogen being a risk factor for vitamin D deficiency in ischemic stroke; besides, it has been reported that high levels of FIB will increase plasma viscosity, which may lead to low cerebral blood flow and impaired perfusion ultimately leading to a higher severity of stroke (Rasyid et al., 2019; Tao et al., 2020). This may explain why NIHSS is more sensitive to the vitamin D deficiency group with high FIB level. Further reports are needed to validate our results and speculations.

Our results should be interpreted with caution given the cross-sectional observational design. A longitudinal study is also needed to validate our hypotheses as well. Another limitation is our study is that our patients were only Chinese patients from the Han ethnicity and majority of our stroke patients were of atherosclerotic etiology, thus, limiting its generalizability to other populations of different ethnic groups. Our study could not perform repeated vitamin D measurement over periods of time to assess the stability of vitamin D. Even so, the measurement of only one vitamin D metabolite. Moreover, studies (Brot et al., 2001) have shown that exposure of sun is an important determinant of 25(OH) D levels; nonetheless, we did not assess the duration and region of sun exposure levels for each participant. Dietary intake (Clements et al., 1987) is another crucial factor that affects vitamin D status; unfortunately, we did not assess and record the dietary intake of each participant.

Although deficient levels of vitamin D are risk factor for ischemic stroke and our current report showed that deficiency of vitamin D is associated with severity of stroke using NHISS. Our results also showed that the risk factors of vitamin D deficiency in stroke patients are the female gender, severity of stroke using NHISS and FIB in all incident stroke patients. Although supplementation of vitamin D has been reported to reduce ischemic stroke and its outcome, reports on this is inconclusive. However, our study urges clinicians to pay close attention to the levels of vitamin D in patients with ischemic stroke.

## Data Availability Statement

The raw data supporting the conclusions of this article will be made available by the authors, without undue reservation.

## Ethics Statement

The studies involving human participants were reviewed and approved by the Ethics Committee of The First Affiliated Hospital of Wenzhou Medical University. The patients/participants provided their written informed consent to participate in this study.

## Author Contributions

HM contributed to conception, data curation, formal analysis, and writing original draft. HZ contributed to the data curation, formal analysis. XL and GH contributed to the data curation. MC contributed to conceptualization and project administration. ZY contributed to funding acquisition, resources, and writing-review and editing. ZW contributed to conceptualization, resources, and writing-review and editing. All authors contributed to the article and approved the submitted version.

## Conflict of Interest

The authors declare that the research was conducted in the absence of any commercial or financial relationships that could be construed as a potential conflict of interest.
